# Modeling Postoperative Nerve Regeneration Using Diffusion MRI: A Preclinical Study of a Novel Mathematical Approach

**DOI:** 10.1002/mus.70110

**Published:** 2025-12-22

**Authors:** Isaac Manzanera Esteve, Ling Yan, Huseyin Karagoz, Ricardo Torres‐Guzman, Sara Chaker, Barite Gutama, Ronald M. Cornely, Benjamin Savitz, Andrew James, Noah Alter, Anthony Hoang, Anvith Reddy, Erin Abott, Ping Wang, Kezia Sharon Christopher, Richard Dortch, Wesley Thayer

**Affiliations:** ^1^ Department of Plastic Surgery Vanderbilt University Medical Center Nashville Tennessee USA; ^2^ Department of Neuroimaging Innovation Center Barrow Neurological Institute Phoenix Arizona USA

**Keywords:** longitudinal model, MRI, nerve recovery, peripheral nerve injury, prognosis

## Abstract

**Introduction/Aims:**

Nerve regeneration after injury must occur in a timely fashion to restore function. Current methods of assessment provide limited information following trauma, resulting in delayed management and suboptimal outcomes. In this study, we evaluated the ability of diffusion magnetic resonance imaging (MRI) and a mathematical model based on the Gompertz function to monitor nerve regeneration after injury and repair.

**Methods:**

Sprague Dawley rats were assigned to two treatment groups (sham = 2, cut, immediate repair = 7), and in vivo diffusion tensor imaging (DTI) was performed every 2 weeks until 12 weeks post‐surgery. Functional recovery was evaluated weekly over the same time period via the sciatic functional index (SFI).

**Results:**

After injury, SFI and DTI‐derived fractional anisotropy (FA) values exhibited similar longitudinal trends and distinctions in both sham and cut/repair (C/R) cohorts. FA values at the distal section displayed the highest correlation with behavioral indices at the region nearest to the injury (*r* = 0.84, *p* < 0.001), followed by FA values at the central section (*r* = 0.82, *p* < 0.001) and the section farthest from the injury (*r* = 0.70, *p* < 0.001).

**Discussion:**

Findings suggest that automated analyses of FA profiles along the nerve may provide insights for distinguishing successful/unsuccessful nerve recovery. This tool, once proven in a larger‐scale study, can provide clinicians with the needed tool to early diagnose nerve recovery and identify cases requiring a second repair surgery.

**Abbreviations**
∆FA: change in FA along the distal portion
AD: axial diffusivity
C/R: cut/repair
DTI: diffusion tensor imaging
DW: diffusion‐weighted
EPI: echo‐planar imaging
FA: fractional anisotropy
FA_0_
: FA value at the top asymptote
FA_IP_
: FA value at the inflexion point
FA_Target_
: FA value at the low asymptote, far from the injury site
MD: mean diffusivity
MRI: magnetic resonance imaging
NLS: nonlinear least squares
PNI: peripheral nerve injury
RD: radial diffusivity
ROI: region of interest
SD: standard deviation
SFI: sciatic functional index
X_IP_
: position of the inflection point

## 1 Introduction

Peripheral nerve injury (PNI) recovery can take many months, especially for more proximal injuries, with associated motor and sensory sequelae that result in chronic pain and disability and hinder quality of life [1]. Up to 40% of surgical repairs of severe peripheral nerve injuries fail, and a secondary repair is required [2]. In addition, there is a 1% loss of achievable sensory and motor function for every 6 days of delay in diagnosis and surgical intervention [3].

In cases involving neurotmesis, the severance of the nerve results in anatomical disruption of axons following Wallerian degeneration expanding from the injury site to the end organ (distal stump) [4]. Connective tissue at the distal stump may remain and form endoneurial tubes in which Schwann cells proliferate and provide the pathway for axonal regeneration taking place at rates varying from 0.5 to 9.0 mm per day [4, 5, 6, 7]. In addition, the nerve recovery rate decreases as the distance between the cell body and axon tip increases [6, 7]. Furthermore, nerve regeneration continues with axonal maturation mechanisms that occur at a slower pace and need to take place before functional recovery can be achieved [4, 7].

Currently, reliable noninvasive tools for monitoring postoperative nerve recovery are lacking, limiting the ability to detect failed repairs and promptly initiate revisional surgery [2]. Diffusion tensor imaging (DTI) has shown promise as a noninvasive tool for identifying nerve injuries and tracking postoperative recovery, and therefore may help fill this current gap. Fractional anisotropy (FA) is a key DTI measure that reports on microstructural tissue anisotropy, with a range of 0–1, in which higher values indicate healthy (densely packed and coherent) nerves and lower values report on reductions in axonal packing and/or coherence related to injury [7, 8, 9, 10, 11]. Our previous studies demonstrated that FA values can differentiate healthy from injured nerves [10, 12]. In addition, we demonstrated that DTI is a viable tool for distinguishing different degrees of nerve injuries, supporting its application in both pre‐ and postoperative monitoring [13, 14].

In this study, we examine FA profiles to characterize the degrees of nerve health along the distal segment of the sciatic nerve (Figure S1A). The complexity of longitudinal FA profiles limits the effectiveness of ROI‐based approaches in fully capturing trends in nerve recovery or regional FA changes. Previous studies have primarily focused on one or two distinct regions distal to the injury site, limiting their ability to fully capture the dynamic and heterogeneous nature of nerve regeneration. To address this limitation, we implement the sigmoid Gompertz function to characterize and identify distal nerve profiles in an automated manner (Figure S1B). The Gompertz function has been successfully implemented as a growth model [15, 16, 17] in multiple areas, including CT scan infection screening [18], COVID‐19 spread [19], wound healing models [20], spinal cord injury recovery [21], and behavioral recovery in rat models after traumatic PNI [22]. We hypothesize that the Gompertz function has the potential to similarly improve our ability to identify and characterize de/regeneration changes along the length of the nerve, which may aid in the diagnosis and subsequent monitoring of recovery after surgical intervention.

## 2 Methods

### 2.1 Ethics Approval

All animal procedures were approved by the Vanderbilt University Medical Center Institutional Animal Care and Use Committee under the Guide for Care and Use of Laboratory Animals to minimize pain and suffering.

### 2.2 Experimental Design

Female Sprague Dawley rats (250–274 g, around 10–12 weeks) were purchased from Envigo (Indianapolis, IN, USA) and housed in ventilated cage racks under standard conditions (12‐h light/dark cycle, 72°F). All rats were given access to food and water ad libitum. Nine rats were randomly assigned either to the group with a full transection and immediate coaptation repair (cut/repair [C/R], *n* = 7) or to the sham surgery cohort (*n* = 2). Magnetic resonance imaging (MRI) scans were performed before surgical intervention and then every 2 weeks until each animal's endpoint (12 weeks) to evaluate sciatic nerve recovery (Figure S2). For comparison, behavioral tests were performed before surgical intervention, 3 days after intervention, and then weekly until each animal's endpoint. Behavioral measurements included the sciatic function index (SFI), as described in previous studies [8, 9]. Each animal was euthanized 12 weeks after surgery with Euthasol (Virbac AH, Fort Worth, TX, USA).

### 2.3 Animal Surgeries

Induction and general anesthesia were performed with a dose of 3 mL/min of 2% isoflurane, and care was taken to minimize the risk of hypothermia. The rat was placed in a prone position, and a 3‐cm skin incision was then made from below the ischial notch parallel to the longitudinal axis of the hind leg. Sciatic nerves were identified and carefully dissected to a clear exposure (shown in Figure S3). The sciatic nerve was freed proximally and distally up to its trifurcation with a length of around 2 cm. Sciatic nerves in the sham group received no intervention. Sciatic nerves in the C/R group were fully transected with a micro‐spring handle scissor at the position around 1 cm proximal to the trifurcation and immediately repaired in an end‐to‐end fashion with interrupted epineurial 9‐0 nylon sutures (Ethicon, Somerville, NJ, USA). Wounds were closed in two layers using a 5‐0 Monocryl suture (Ethicon). After surgery, all animals were carefully monitored for any adverse anesthetic effects and provided with daily injections of meloxicam (4 mg/kg) for 3 days postoperatively.

### 2.4 MRI Protocol

Rats were induced into anesthesia using 4% isoflurane in oxygen concentration. Once the rat was induced via induction chamber, it was moved to a semicircular custom‐made Plexiglas cradle (6.4 mm ID, 7 mm OD), and anesthesia was maintained via nose cone using 1.5% isoflurane in oxygen for maintenance throughout the duration of the MRI scan. Rats were placed in the cradle in a right lateral recumbent position with a small blanket to maintain rodent body temperature (Figure S4). A respiration pillow sensor and a temperature probe were attached to each anesthetized rat to continuously monitor respiration and body temperature, ensuring that the animals remained in a stable physiological condition during MRI acquisition. Tape was gently wrapped around the left foot and attached to the inside of the Plexiglas cradle to ensure full extension of the sciatic nerve and consistent positioning of the sciatic nerve across rats and time points [9, 23]. Diffusion‐weighted (DW) MRI data were then acquired at a controlled temperature of 33°C using a 7‐T, 16 cm bore Bruker Biospec console (Rheinstetten, Germany). For transmission, a 72‐mm inner diameter quadrature radio‐frequency coil (089/072 QSN TR AD Brunker, Rheinstetten, Germany) was used, and for reception, a rat surface coil array (ARR 300 1H R.BR. 2 × 2 LIN RO AD Brunker, Rheinstetten, Germany) was placed above the injured leg. Images were acquired with a three‐dimensional DW echo‐planar imaging (EPI) sequence with the following parameters: field‐of‐view = 32 × 32 × 38.4 mm^3^, resolution = 0.32 × 0.32 × 0.8 mm^3^, TE/TR = 26/3000 ms, gradient pulse duration/diffusion time (δ/Δ) = 3/9 ms, *b* = 1000 s/mm^2^, 30 diffusion directions, number of averaged excitations = 2, three non‐DW (b0) images, and scan time = 1 h 3 min.

### 2.5 MRI Analysis

Diffusion tensors were estimated on a voxel‐wise basis using the ExploreDTI Toolbox in MATLAB (Mathworks, Natick, MA, USA) using weighted linear least‐squares regression, from which FA was estimated [9]. Regions of interest (ROIs) were then drawn manually in each axial cross‐section of the nerve to calculate the mean (and standard deviation [SD]) slice‐wise FA values. Each slice along the length of the sciatic nerve was then manually classified based on a combination of the FA map (Figure S5B) and anatomical features like nerve diameter (Figure S5C). The region proximal to the injury generally exhibited higher, consistent FA values (green values in Figure S5D). In contrast, the area corresponding to the injury was characterized by a sharp decline in FA values (red values in Figure S5D), while the distal region was characterized by further reductions in both FA values (Figure S5D) and nerve diameters. Since nerves in sham cohorts do not have a C/R injury (Figure S3), they do not have to go over the degeneration/regeneration mechanisms, and consequently, they do not have, strictly speaking, a distal region. However, sections closer to the target muscle of approximately 15 mm were chosen as the distal regions to match the C/R cohort counterparts.

For each rat and time point, FA profiles of the distal site were isolated (Figure S5E) and fitted to a Gompertz function (Figure S5F). Mathematically, the Gompertz function is described by Equation (1) below and the following parameters: (i) FA value at the top asymptote, close to the injury site, or FA_0_; (ii) FA value at the low asymptote, far from the injury site, or FA_Target_; (iii) FA value at the inflexion point, or ${\mathrm{FA}}_{\mathrm{IP}}={\mathrm{FA}}_{0}+∆\mathrm{FA}/e$; (iv) the position of the inflection point along the sciatic nerve, or X_IP_; and (v) the change in FA along the distal portion of ∆FA

(1) $$\mathrm{FA}\left(x\right)={\mathrm{FA}}_{0}+∆\mathrm{FA}\times e^{-e^{\left(-b\times \left(x-x_{\mathrm{IP}}\right)\right)}}.$$

### 2.6 Behavioral Testing

Before surgery, habituation trials were performed to allow animals to navigate an inclined beam into their home cage without hesitation. Each hind limb was inked, and animals walked along a flat wooden runway (8‐cm wide, 100‐cm long) until six consecutive footprints (three from each limb) were recorded on a white paper strip placed along the runway. This setup encouraged consistent traversal while capturing high‐contrast footprints for analysis. We closely monitored ambulation to ensure all rats ascended the inclined beam in a comparable manner and velocity, thereby minimizing variability in print length and potential inaccuracies in SFI measurements [24]. The following measurements were taken from the prints: normal toe spread (NTS), normal print length (NPL), normal intermediary toe spread (NIT), experimental toe spread (ETS), experimental print length (EPL), and experimental intermediary toe spread (EIT). SFI scores were then calculated using the following formula [24, 25].

(2) $$\mathrm{SFI}=-38.3\left(\frac{\mathrm{EPL}-\mathrm{NPL}}{\mathrm{NPL}}\right)+109.5\left(\frac{\mathrm{ETS}-\mathrm{NTS}}{\mathrm{NTS}}\right)+13.3\left(\frac{\mathrm{EIT}-\mathrm{NIT}}{\mathrm{NIT}}\right)$$

We obtained measures of SFI 3 days post‐injury and weekly for 12 weeks (Figure S2), as described in previous studies [22]. A single assessment for each behavioral measurement was collected in each rat at each time point to avoid overstimulating the animal, which would result in poor performance on these tasks. Assessments were performed by the same individual throughout the study to ensure consistency. Behavioral data were grouped by injury type and time point to provide insight into the temporal dynamics of the recovery process with each cohort.

### 2.7 Statistical Analysis

All statistical analyses were performed using R, version 4.3.1. FA profiles were obtained by calculating the mean ± SD of the ROI for each slice. FA profiles were then fit using the nonlinear regression model (NLS library—stats package in R, version 3.6.1), using the initial values and boundary constraints provided in Table S1 [26, 27]. These approaches provided Gompertz parameter estimates and associated uncertainties. In addition, uncertainty values for SFI measurements were calculated using standard error propagation of Equation (2), where the uncertainty of each variable was determined based on the sensitivity of the measurement to EPL, NPL, ETS, NTS, EIT, and NIT.

To evaluate the longitudinal associations between SFI and the derived parameters (FA_0_, FA_IP_, ∆FA, FA_Target_, and X_IP_), Pearson correlation coefficients and corresponding *p* values were computed, with Bonferroni correction applied to control for multiple statistical comparisons (corrected *p* < 0.05 deemed statistically significant).

To account for the relatively small sample size, a repeated measures ANOVA was implemented (AOV function—stats package in R, version 3.6.1). This approach fit a linear model relating SFI to the FA‐derived parameters (FA_0_, FA_IP_, ∆FA, FA_Target_, and X_IP_) across repeated measures, with each animal included as an error term to account for within‐subject correlations. This allowed us to assess whether changes in FA‐derived parameters over time were systematically associated with SFI at the group level [28].

## 3 Results

### 3.1 SFI

**FIGURE 1:**
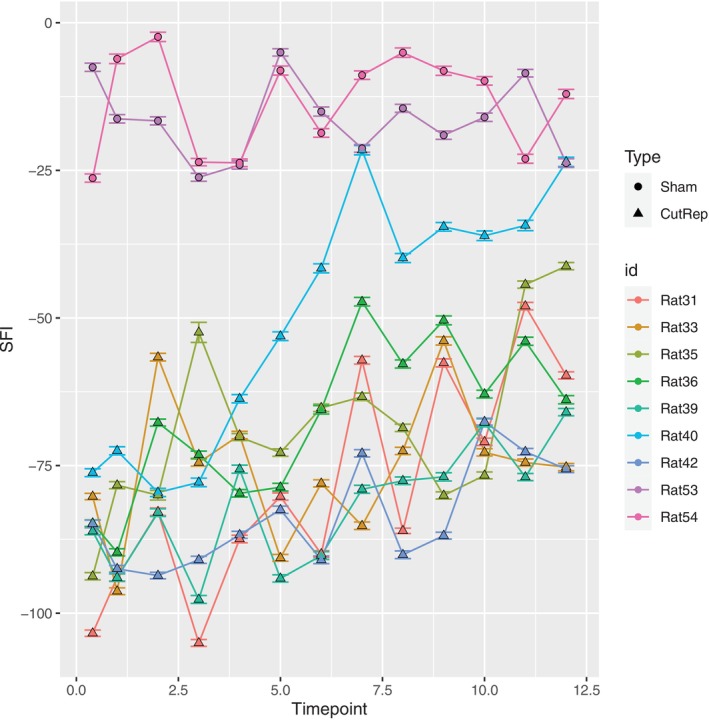
Longitudinal evolution of SFI parameters for Sham cohort (circles) and cut/repair (triangles). Time points are 3 days and then weekly after surgery. SFI values and lines are color‐coded for each sample.

Temporal evolution of measured (±error) SFI values for the C/R and sham cohorts is shown in Figure 1. Sham rats remained within the −30 and 0 range across all time points. In contrast, the C/R rats began with SFI values between −75 and −115 immediately after surgery, which gradually increased over time at variable rates. By Week 12, SFI values ranged from −75 to −25 in this cohort.

### 3.2 FA

**FIGURE 2:**
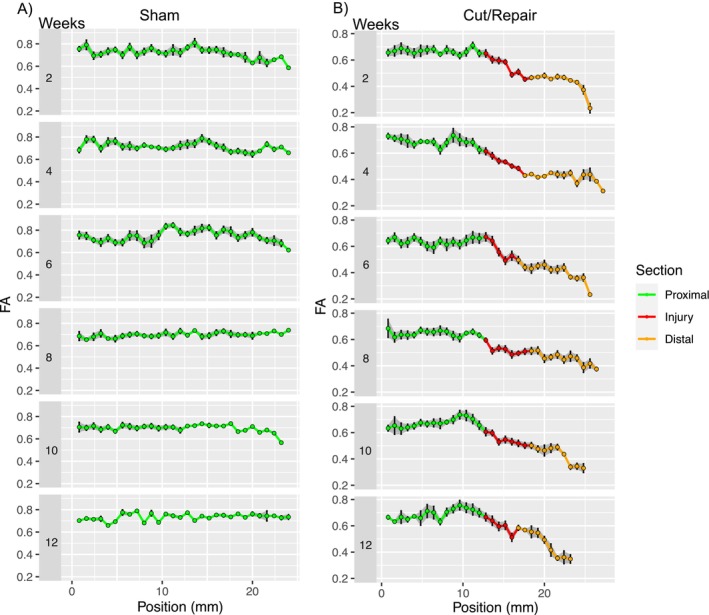
Representative FA profiles and error bars of a sham (A) and a cut/repair (B) sciatic nerve. Profiles were measured at 2, 4, 6, 8, 10, and 12 weeks after repair surgery. Green points correspond to the proximal region, red points indicate the injury region, and orange points represent the distal region.

Figure 2 shows the evolution of a representative mean (±SD) FA profile in a rat following a sham (Figure 2A) and a C/R (Figure 2B) surgery of the sciatic nerve across proximal, injury, and distal regions. Across all time points, the mean FA value for the entire profile was 0.713 ± 0.003. Meanwhile, across all time points, we observed lower overall FA values at the injury section (FA = 0.55 ± 0.01) and distal sites (FA = 0.433 ± 0.009) compared to the proximal site (FA = 0.666 ± 0.004), which likely indicates the degeneration (and subsequent regeneration) of axons after the injury and repair. However, due to the complex changes in the mean FA values along the length of the distal site, it is difficult to fully capture the evolution of distal FA values across the 12‐week postsurgical period using simple ROI‐based approaches.

**FIGURE 3:**
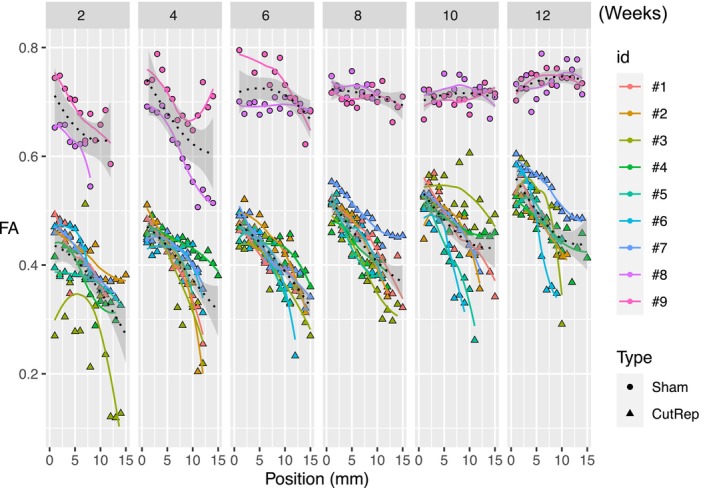
Representation of FA profiles of the distal site for sham nerves (circles) and cut/repair (triangles). Time points are 2, 4, 6, 8, 10, and 12 weeks post‐surgery. FA values and Gompertz function fit lines are color‐coded for each sample. Mean FA values of each cohort (sham and cut/repair) are represented as a black dotted line with standard error included in the shape of a ribbon in light gray color.

Distal FA profiles of the sciatic nerves are depicted in Figure 3. Here, the differences between C/R and sham nerve distal FA values can be easily seen. The distal FA values in the C/R cohort were lower than those in the sham cohort at all time points, which is consistent with the axonal distribution in this cohort. Over time, we observed an overall increase in the FA values for the C/R cohort, consistent with our behavioral recovery (as detailed below) and likely related to nerve regeneration.

### 3.3 Validation

**FIGURE 4:**
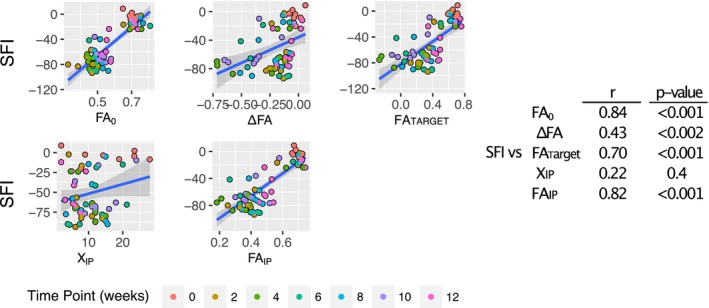
Correlations between SFI and Gompertz‐based FA parameters. (A) Graphical representation of the correlations between the behavior variable SFI and the FA‐derived parameters FA_0_, ∆FA, FA_Target_, X_IP_, and FA_IP_. Plots are color‐coded by time points, and 0 represents measurements before the injury. (B) Table indicating Pearson's correlation coefficients and *p* values.

Our model targets nerve profiles as an additional source of information and more fully captures nerve recovery evolution. In addition, fitting a smooth function to our data removes sensitivity to noise across different slices. As seen in Figure S1, applying the Gompertz function segments the distal FA profile into three regions described by five parameters. Each parameter was calculated for every rat and time point and subsequently correlated with the longitudinal behavioral SFI data using the Gompertz‐based FA model (Figure 4). Of the FA parameters obtained from the model, the strongest correlation between FA values and SFI was for FA_0_, FA_IP_, and FA_Target_. In contrast, ∆FA correlated poorly with SFI data. In addition, the study indicated no statistically significant correlation between X_IP_ and SFI data.

A significant association was found between SFI and FA_0_ (*F*(1,53) = 110.2, *p* < 0.0001), as well as between SFI and FA_Target_ (*F*(1,53) = 34.72, *p* < 0.0001). The variance between SFI and ∆FA was also significant (*F*(1,53) = 10.3, *p* < 0.001). In contrast, associations with FA_IP_ (*F*(1,53) = 2.68, *p* < 0.05) and X_IP_ (*F*(1,53) = 3.56, *p* < 0.1) were weaker, with only FA_IP_ reaching conventional statistical significance.

## 4 Discussion

Using a Gompertz‐based mathematical model, we identified three primary regions within the distal sciatic nerve described by the FA values (FA_0_, FA_Target_, and FA_IP_) that strongly correlate with behavioral recovery (i.e., SFI). FA has been previously shown to be a reliable biomarker of nerve integrity, capable of identifying and monitoring nerve recovery [29, 30]. Decreases in FA values have been associated with demyelination and loss of axonal structure, while increases in FA values have been proven to correlate with nerve regeneration [14, 30, 31, 32, 33]. In this study, we evaluated a novel mathematical model that may offer an approved approach to extract and analyze the evolution of FA values along the distal portion of the nerve following trauma and surgical repair.

Nerve regeneration begins with axonal growth and is followed by the slower axonal maturation mechanisms (remyelination, axonal enlargement, and establishment of connections with the end organ), which must occur before functional recovery can be achieved. By 2 weeks post‐injury, we expect axonal tip growth to have reached approximately 14 mm in length, based on findings from previous studies [6, 7]. Given that the distal segment analyzed in this study was approximately 15 mm in length, we postulate that our observed regeneration within this region (after 2 weeks) was primarily driven by axonal maturation processes—specifically, (i) remyelination and (ii) axonal enlargement. These multiple mechanisms, progressing from the injury site to the axon tip, may explain why FA profiles of the distal site exhibit complex nonlinear sigmoid profiles.

Prior research on PNI has largely relied on FA measurements at the distal site to evaluate post‐injury recovery [10, 13, 34]. In some studies, FA values were reported from a single defined region [14, 35], while others measured the entire distal segment [36]. In contrast, fitting each distal section's profile to a Gompertz function results in two key advantages: (i) automated segmentation of the profile into three regions (Figures S1B and S5F), which eliminates the potential for user‐introduced ROI selection bias; and (ii) estimation of the FA value for each section by weighting all points from the distal profile, thereby reducing sensitivity to noise that would arise if only a small group of adjacent slices were analyzed. Likewise, the observed decline in FA values along the distal segment in the C/R cohort suggests a progressive decrease in axonal organization and integrity with increasing distance from the injury site, likely reflecting the presence of thinner axons and reduced myelin thickness [31, 32, 33, 34, 37, 38].

The result of this process is an FA value for each segment (FA_0_, FA_IP_, and FA_Target_), a measurement of the decrease in FA (∆FA), and the position where the slope is maximal (X_IP_). Figure 2 illustrates the changes in FA along the entire nerve profile during recovery. The data reveal a complex pattern of FA evolution at the distal site (orange), with distinct variations depending on both anatomical region and time point. In Figure 3, sham profiles at 2 and 4 weeks depicted a substantial variation of the FA values along the distal site, while C/R FA profiles show a comparable patternbut with lower overall FA values. Between 6 and 12 weeks, sham FA profiles were relatively flat and consistent. On the other hand, FA profiles of the C/R cohort between 6 and 12 weeks were generally characterized by a complex nonlinear reduction of FA values further from the injury, with a notable overall increase in the FA values with time. Overall, regardless of the time point post‐injury, FA values in the C/R group remained significantly lower than those in the sham group, indicating that FA can reliably distinguish injured nerves from healthy ones at all stages of recovery.

As shown in Figure 4, FA measurements closest to the injury site, FA_0_, showed the strongest correlation with behavioral outcomes, followed by the middle section, FA_IP_. The weakest correlation was observed in the region farthest from the injury FA_Target_. These results suggest that Gompertz‐based FA values (FA_0_, FA_IP_, and FA_Target_) may be promising candidates to analyze nerve recovery. On the other hand, correlations for the location of the inflexion point (X_IP_) and the difference in FA values along the distal segment (∆FA) were weak, suggesting that these parameters may be poor biomarkers of nerve recovery.

Notice cases where low correlation values are accompanied by low *p* values, as seen in the case of ∆FA and SFI. This occurs when one of the variables carries substantial uncertainty. In our study, the relatively large uncertainty of ∆FA, stemming from the propagation of errors from both FA_0_ and FA_Target_, likely explains the modest correlation value despite the low *p* value. Overall, these findings suggest a strong relationship between FA‐based measures, FA_0_, and FA_Target_, which may reflect axonal maturation and behavioral outcomes.

Although promising, this study has several limitations. First, the extended scan time required for high‐resolution DTI imaging (> 1 h) limited the size of our study population, and future larger population studies will be required to avoid overgeneralization. Moreover, this study focused on FA profiles. Additional DTI parameters, such as (i) mean diffusivity (MD), (ii) axial diffusivity (AD), and (iii) radial diffusivity (RD), and other myelin content sensitive techniques like MTR, may complement information related to nerve injury and regeneration [39, 40]. However, these variables are beyond the scope of this work and will be included in future studies. Further research is needed to explore how additional DTI parameters and MTR measurements can be integrated into the proposed mathematical model for assessing nerve recovery. Application of MTR may be of particular value clinically, as these scans can typically be performed using shorter, higher‐resolution scans than DTI. In addition, this study did not include a crush injury or autograft cohort; future work will aim to evaluate different types of nerve injuries. While previous studies have applied DTI and MTR to analyze peripheral nerves in patients [39, 40], future longitudinal studies will specifically focus on applying DTI, MTR, and the proposed mathematical modeling framework to track nerve recovery after PNI, with the ultimate goal of predicting clinical outcomes.

## 5 Conclusion

FA values along the sciatic nerve exhibit nonlinear profiles that evolve over time, complicating the analysis of nerve regeneration. Applying the Gompertz function to the FA values of the distal site results in an unbiased segmentation into three regions with their corresponding FA values (FA_0_, FA_IP_, and FA_Target_). The resulting FA values strongly correlate with SFI behavioral data, and the correlations decrease as the distance from the injury site increases. These findings support the use of Gompertz‐modeled FA parameters as potential biomarkers for evaluating and predicting postoperative peripheral nerve regeneration.

## Author Contributions

**Isaac Manzanera Esteve:** conceptualization, investigation, funding acquisition, writing – original draft, methodology, validation, visualization, writing – review and editing, software, formal analysis, project administration, data curation, supervision, and resources. **Ling Yan:** investigation, methodology, writing – review and editing, supervision, and resources. **Huseyin Karagoz:** investigation, writing – review and editing, supervision, methodology. **Ricardo Torres‐Guzman:** investigation, writing – review and editing, and supervision. **Sara Chaker:** investigation, writing – review and editing, formal analysis. **Barite Gutama:** investigation, writing – review and editing, supervision. **Ronald M. Cornely:** writing – review and editing, investigation. **Benjamin Savitz:** investigation, writing – review and editing. **Andrew James:** investigation, writing – review and editing. **Noah Alter:** investigation, writing – review and editing. **Anthony Hoang:** investigation, writing – review and editing. **Anvith Reddy:** investigation, writing – review and editing. **Erin Abott:** writing – review and editing, investigation. **Ping Wang:** writing – review and editing, supervision. **Kezia Sharon Christopher:** writing – review and editing, supervision. **Richard Dortch:** conceptualization, investigation, funding acquisition, methodology, writing – review and editing, visualization, and supervision. **Wesley Thayer:** conceptualization, investigation, funding acquisition, methodology, writing – review and editing, supervision.

## Funding

This study was funded by MRI Biomarkers of Traumatic Peripheral Nerve Injury and Repair: Validation and Multisite Application (Award Number W81XWH‐22‐1‐048) and Diffusion MRI Biomarkers of Peripheral Nerve Trauma (Grant Number: 5R61NS127268‐02).

## Ethics Statement

The authors state that they have obtained appropriate institutional review board approval and have followed the principles outlined in the Declaration of Helsinki for all human or animal experimental investigations. The Institutional Animal Care & Use Committee (IACUC) approved protocol M2200006‐01. The protocol M2200006‐00 was originally approved before May 2022, and the most recent review and approval were in January 2025.

## Conflicts of Interest

The authors declare no conflicts of interest.

## Supporting information


**Figure S1:** Parameters of fitted function and relevance to injured nerve. Graphical cross‐sections of nerve health based on axonal density, axonal diameter, myelin thickness and extra‐axonal volume (A) Top are arranged by location relative to injury (proximal, distal close to or far from injury) and by time point t1, t2 and t3 corresponding to ~4,8 and 12 weeks. Representative FA profiles at different time‐points are color‐coded by FA value. FA profiles are segmented into Proximal, Injury and Distal regions (black arrows). Distal region (sigmoid shaped) is divided in three smaller sections (distal close to injury, distal far from injury and a transition section) delimited by thin black segments. Color‐coded arrows relate nerve cross sections with sections of the FA profiles and time‐points. Gompertz fit (B) of FA profile at 10 weeks (black line) describes the distal region with the parameters (blue) FA_0_, FA_Target_, FA_IP_, ∆FA and X_I_.

**Figure S2:** A scheme illustrating the timeline for MRI scans on the sciatic nerve and behavioral sciatic function index (SFI) measurements.

**Figure S3:** Sciatic nerve with indication of location where cut and repair surgery takes place.

**Figure 4.** Positioning of rat in cradle without (left) and with (right) surface coil on top.

**Figure 5.** Graphical representation of the MRI workflow implemented in this study from data acquisition to FA parameters extraction. (A) Anatomical and diffusion MRI scans. Analysis of diffusion‐weighted EPI results in (B) FA maps and (C) anatomical b0 analysis allows for identifying and classifying the nerve based on its shape. Anatomical feature extraction divides the neve into three regions: Proximal, Injury, and Distal. (D) FA profiles extracted from FA maps (D) are color‐coded based on the previous regions Proximal (green), Injury (red), and Distal (orange). (E) Extraction of Distal region from the complete FA profile. (F) Fitting the Gompertz function to the FA profile of the distal region results in multiple FA parameters that describe the profile. FA_0_ indicates the FA value of the upper asymptote corresponding to the region closer to the injury, FA_Target_ denotes the FA value of the lower asymptote or region closer to the muscle end target, ∆FA is the difference between the previous parameters. FA_IP_ is the FA value at the inflection point X_IP_ located in the middle section.

**Table S1:** Initial values used for the nonlinear regression models (nls).

## Data Availability

The data that support the findings of this study are available from the corresponding author upon reasonable request.
