# Supplementary material for: Modeling Postoperative Nerve Regeneration Using Diffusion MRI: A Preclinical Study of a Novel Mathematical Approach
Source: Muscle Nerve. 2025 Dec 22;73(2):346–54. doi: 10.1002/mus.70110 (PMC12803670; doi:10.1002/mus.70110)
Supplement: Supplementary file 1 — Figure S1: Parameters of fitted function and relevance to injured nerve. Graphical cross‐sections of nerve health based on axonal density, axonal diameter, myelin thickness and extra‐axonal volume (A) Top are arranged by location relative to injury (proximal, distal close to or far from injury) and by time point t1, t2 and t3 corresponding to ~4,8 and 12 weeks. Representative FA profiles at different time‐points are color‐coded by FA value. FA profiles are segmented into Proximal, Injury and Distal regions (black arrows). Distal region (sigmoid shaped) is divided in three smaller sections (distal close to injury, distal far from injury and a transition section) delimited by thin black segments. Color‐coded arrows relate nerve cross sections with sections of the FA profiles and time‐points. Gompertz fit (B) of FA profile at 10 weeks (black line) describes the distal region with the parameters (blue) FA0, FATarget, FAIP, ∆FA and XI. [file MUS-73-346-s004.pdf]

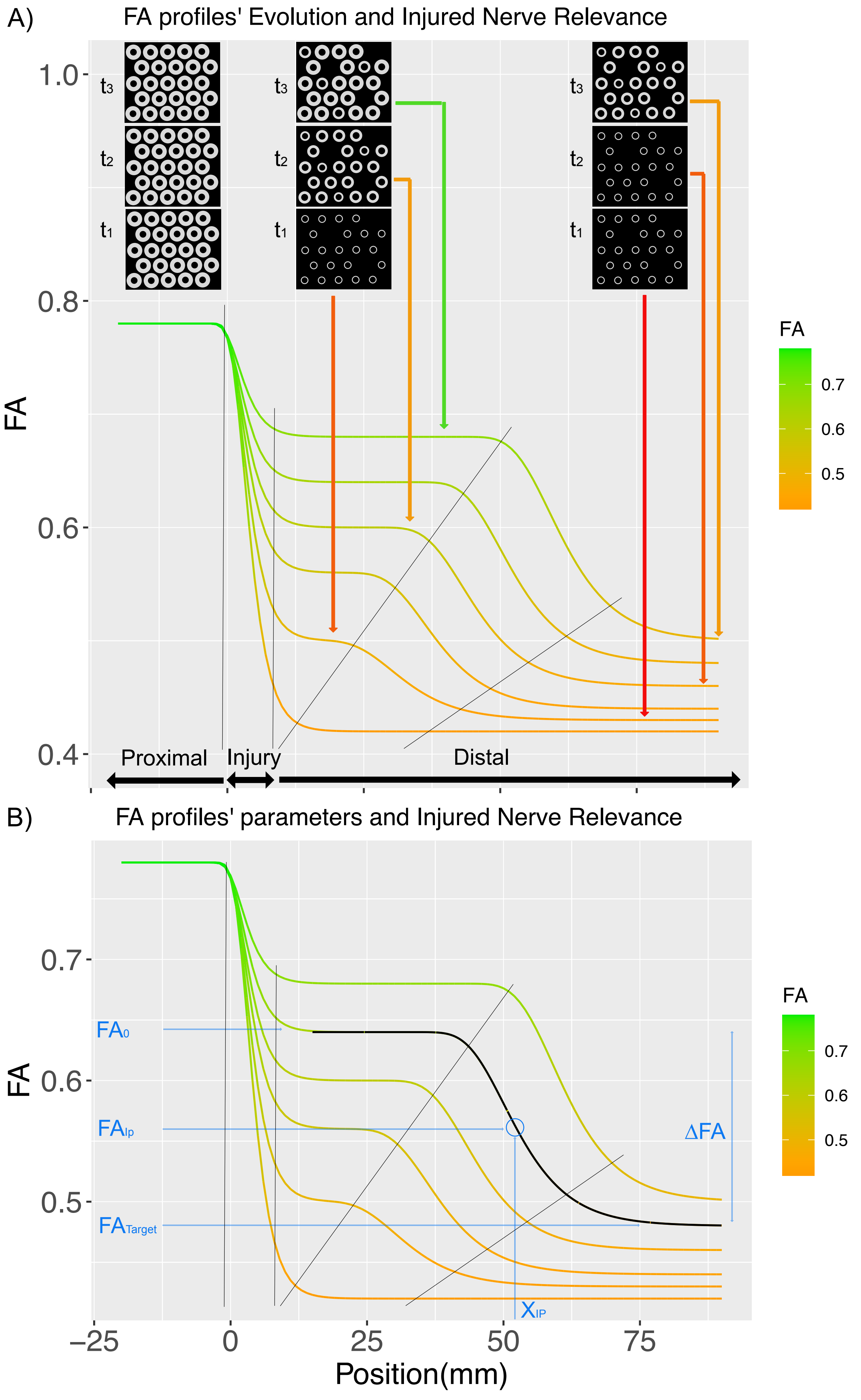

A)
FA profiles' Evolution and Injured Nerve Relevance
t3
t2
t1
FA
1.0
0.8
0.6
0.4
Proximal
Injury
Distal
0.7
0.6
0.5
B)
FA profiles' parameters and Injured Nerve Relevance
FA0
FAIp
FATarget
XIP
ΔFA
0.7
0.6
0.5
–25
0
25
50
75
Position(mm)
