# Supplementary figures and images for: Modeling Postoperative Nerve Regeneration Using Diffusion MRI: A Preclinical Study of a Novel Mathematical Approach
Source: Muscle Nerve. 2025 Dec 22;73(2):346–54. doi: 10.1002/mus.70110 (PMC12803670; doi:10.1002/mus.70110)

Behavioral

MRI

Surgery

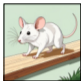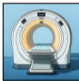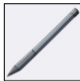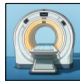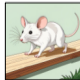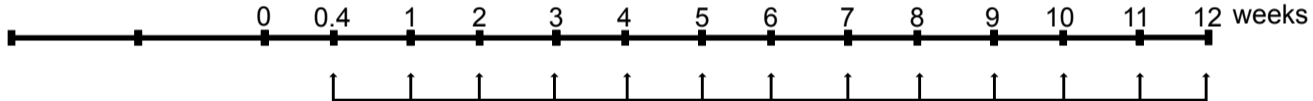

Supplement: Supplementary file 2 — Figure S2: A scheme illustrating the timeline for MRI scans on the sciatic nerve and behavioral sciatic function index (SFI) measurements. [file MUS-73-346-s003.pdf]

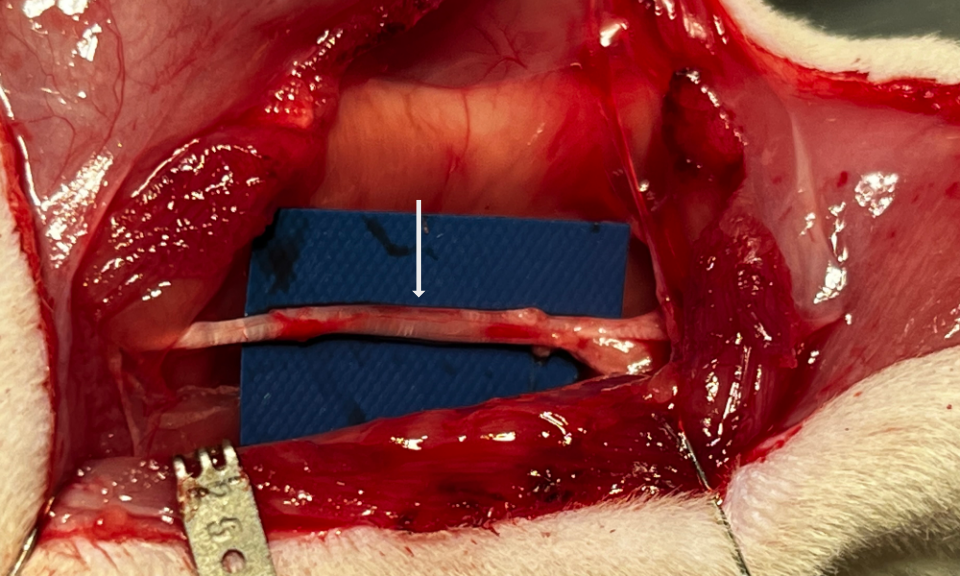

Supplement: Supplementary file 3 — Figure S3: Sciatic nerve with indication of location where cut and repair surgery takes place. [file MUS-73-346-s001.pdf]

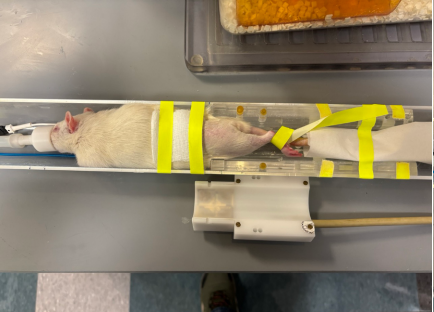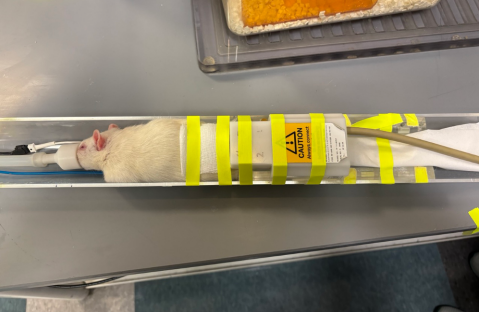

Supplement: Supplementary file 4 — Figure 4. Positioning of rat in cradle without (left) and with (right) surface coil on top. [file MUS-73-346-s005.pdf]
