# Supplementary material for: Modeling Postoperative Nerve Regeneration Using Diffusion MRI: A Preclinical Study of a Novel Mathematical Approach
Source: Muscle Nerve. 2025 Dec 22;73(2):346–54. doi: 10.1002/mus.70110 (PMC12803670; doi:10.1002/mus.70110)
Supplement: Supplementary file 5 — Figure 5. Graphical representation of the MRI workflow implemented in this study from data acquisition to FA parameters extraction. (A) Anatomical and diffusion MRI scans. Analysis of diffusion‐weighted EPI results in (B) FA maps and (C) anatomical b0 analysis allows for identifying and classifying the nerve based on its shape. Anatomical feature extraction divides the neve into three regions: Proximal, Injury, and Distal. (D) FA profiles extracted from FA maps (D) are color‐coded based on the previous regions Proximal (green), Injury (red), and Distal (orange). (E) Extraction of Distal region from the complete FA profile. (F) Fitting the Gompertz function to the FA profile of the distal region results in multiple FA parameters that describe the profile. FA0 indicates the FA value of the upper asymptote corresponding to the region closer to the injury, FATarget denotes the FA value of the lower asymptote or region closer to the muscle end target, ∆FA is the difference between the previous parameters. FAIP is the FA value at the inflection point XIP located in the middle section. [file MUS-73-346-s002.pdf]

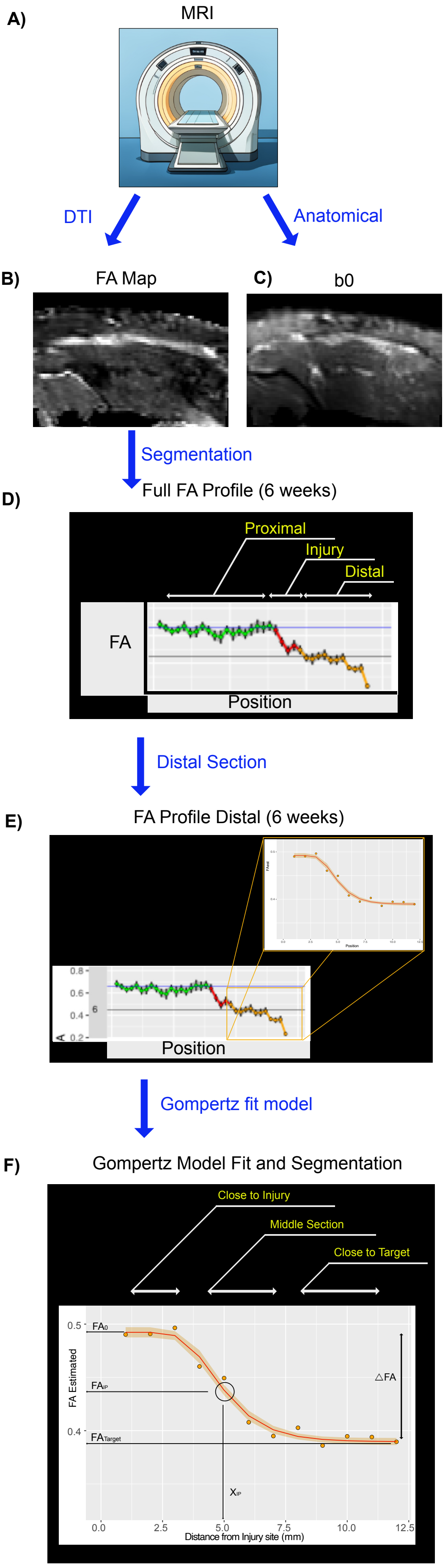
A)
MRI
DTI
Anatomical
B)
FA Map
C)
b0
Segmentation
D)
Full FA Profile (6 weeks)
Proximal
Injury
Distal
FA
Position
Distal Section
E)
FA Profile Distal (6 weeks)
Position
Gompertz fit model
F)
Gompertz Model Fit and Segmentation
Close to Injury
Middle Section
Close to Target
FA Estimated
FA0
FAIP
FATarget
△FA
XIP
0.5
0.4
0.0
2.5
5.0
7.5
10.0
12.5
Distance from Injury site (mm)
