# Supplementary material for: Modeling Postoperative Nerve Regeneration Using Diffusion MRI: A Preclinical Study of a Novel Mathematical Approach
Source: Muscle Nerve. 2025 Dec 22;73(2):346–54. doi: 10.1002/mus.70110 (PMC12803670; doi:10.1002/mus.70110)
Supplement: Supplementary file 6 — Table S1: Initial values used for the nonlinear regression models (nls). [file MUS-73-346-s006.docx]

Supplemental Table 1. Initial values used for the nonlinear regression models (nls).

|  | FA_0_ | ∆FA | b | X_IP_ |
| --- | --- | --- | --- | --- |
| Starting | 0.55 | -0.1 | 0.6 | 11 |
| Upper limit | 0.8 | 1 | 10 | 40 |
| Lower limit | 0.3 | -5 | 0.01 | 2 |
|  |  |  |  |  |
| Maximum number iterations = | | 1x10^8^ | Tolerance = | 1x10^-6^ |
